# Actively Tunable Terahertz Switches Based on Subwavelength Graphene Waveguide

**DOI:** 10.3390/nano8090665

**Published:** 2018-08-26

**Authors:** Zhongyi Guo, Xiaoru Nie, Fei Shen, Hongping Zhou, Qingfeng Zhou, Jun Gao, Kai Guo

**Affiliations:** School of Computer and Information, Hefei University of Technology, Hefei 230009, China; niexiaoru9391@163.com (X.N.); shenfei@hfut.edu.cn (F.S.); ciangela@hfut.edu.cn (H.Z.); enqfzhou@hfut.edu.cn (Q.Z.); gaojun@hfut.edu.cn (J.G.)

**Keywords:** graphene, waveguide, optical switches

## Abstract

As a new field of optical communication technology, on-chip graphene devices are of great interest due to their active tunability and subwavelength scale. In this paper, we systematically investigate optical switches at frequency of 30 THz, including Y-branch (1 × 2), X-branch (2 × 2), single-input three-output (1 × 3), two-input three-output (2 × 3), and two-input four-output (2 × 4) switches. In these devices, a graphene monolayer is stacked on the top of a PMMA (poly methyl methacrylate methacrylic acid) dielectric layer. The optical response of graphene can be electrically manipulated; therefore, the state of each channel can be switched ON and OFF. Numerical simulations demonstrate that the transmission direction can be well manipulated in these devices. In addition, the proposed devices possess advantages of appropriate ON/OFF ratios, indicating the good performance of graphene in terahertz switching. These devices provide a new route toward terahertz optical switching.

## 1. Introduction

Actively tunable switches are among the key devices in optical communication systems and integrated circuits. A main category of switches in previous literature is based on waveguide structures, which is suitable for on-chip application. In this category, the switching mechanism relies on the modulation of optical properties of waveguide composite materials, such as electro- and thermo-optical materials [1,2,3]. One of the desired features of a switch is miniaturization, requiring deep subwavelength scales of both the waveguide structure and concentration of light. To this end, surface plasmon (SP) waveguides and metamaterials (metasurface) have been investigated and proposed to obtain optical switching devices [4,5,6,7,8]. However, metals also bring large intrinsic losses and metasurfaces are still bulky, restricting the practical application of SP switching.

Graphene has emerged as a fascinating alternative for metal due to its flexible tunability and high confinement of light with relatively low loss in the terahertz region [9,10,11]. By tuning the Fermi level, graphene may behave like a thin metal and strongly interact with incident light, thus motivating SPs along the surface of the graphene sheet. It can harness, squeeze, and manipulate electromagnetic waves via simply applying an external applied voltage, leading to the manipulation of graphene SPs at the graphene/dielectric interface [12,13]. These unique properties make graphene a suitable candidate for tunable and compact terahertz switching devices. Yarahmadi et al. proposed a subwavelength terahertz switch based on a Y-branch graphene/gold hybrid structure [14]. They further reported a graphene-based plasmonic waveguide, performing as a switch or an AND/OR logic gate at a frequency of 6 THz [15]. Wu et al. further designed all-optical logic devices based on graphene SPs to achieve six different basic logic gates by utilizing interference between the SPs wave in different channels [16]. Recently, Peng et al. proposed a general theoretical model to obtain the optimal solution for a linear-optical logic gate [17]. However, it is still urgent to develop terahertz switches with multiple-input multiple-output (MIMO) due to the requirement of the communication system to have a large capacity.

In this study, we systematically investigated different types of optical switches at a frequency of 30 THz, including Y-branch (1 × 2), X-branch (2 × 2), single-input three-output (1 × 3), two-input three-output (2 × 3), and two-input four-output (2 × 4) switches. These devices are based on graphene waveguides, consisting of a graphene monolayer, PMMA (poly methyl methacrylate methacrylic acid) interlayer, and silicon substrate. Numerical simulation results demonstrate that the effective optical response of the proposed waveguide structure can be tuned in the subwavelength scale via an external gate voltage, therefore achieving ON/OFF or “1/0” states. Based on this result, we further designed terahertz optical switches to realize several logic gates with the aid of an external gate voltage. We show that the transmission direction can be well manipulated, demonstrating the superior performance of graphene in terahertz switching.

## 2. Theory and Simulation Method

**Figure 1:**
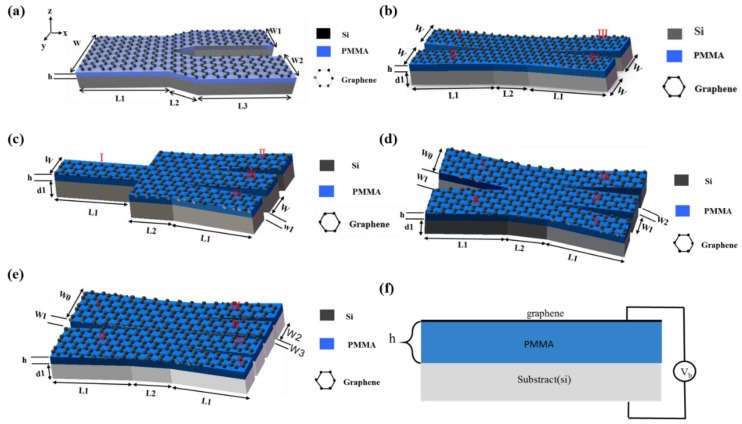
(**a**) Schematic of the Y-branch switch structure. *W* = 800 nm, *h* = 50 nm, *L*_1_ = 500 nm, *L*_2_ = 1000 nm, *L*_3_ = 1500 nm, *W*_1_ = *W*_2_ = 400 nm; (**b**) Schematic of the X-branch switch structure. *W* = 400 nm, *h* = 50 nm, *L*_1_ = 1000 nm, *L*_2_ = 500 nm, *d*_1_ = 200 nm; (**c**) Schematic of the single-input three-output structure. *W* = 400 nm, *W*_1_ = 200 nm, *h* = 50 nm, *d*_1_ = 200 nm, *L*_1_ = 1000 nm, *L*_2_ = 500 nm; (**d**) Schematic of the two-input three-output structure. *W*_0_ = 600 nm, *W*_1_ = 400 nm, *W*_2_ = 200 nm, *h* = 50 nm, *d*_1_ = 200 nm, *L*_1_ = 1000 nm, *L*_2_ = 500 nm; (**e**) Schematic of the two-input four-output structure. *W*_0_ = 800 nm, *W*_1_ = 200 nm, *W*_2_ = 400 nm, *W*_3_ = 100 nm, *h* = 50 nm, *d*_1_ = 200 nm, *L*_1_ = 1000 nm, *L*_2_ = 500 nm; (**f**) Schematic illustration of applying the bias voltage.

Figure 1a–e schematically show the Y-branch (1 × 2), X-branch (2 × 2), single-input three-output (1 × 3), two-input three-output (2 × 3), and two-input four-output (2 × 4) terahertz switches, respectively. The switches consist of graphene, PMMA, and silicon substrate, supporting graphene-based SPs. In this structure, the thickness of the silicon substrate and PMMA are set at 200 nm and 50 nm, respectively. Meanwhile, the graphene monolayer is treated as an ultrathin film layer with a thickness of Δ = 1 nm [18,19,20]. The permittivity of silicon and PMMA is 11.7 and 2.25, respectively. The relative permittivity of graphene is set as:

(1) $$\varepsilon_{g}=1+\frac{i\sigma_{g}}{\omega \varepsilon_{0}\Delta }$$

where $\varepsilon_{0}$ is the permittivity in vacuum. In terahertz range, the surface conductivity $\sigma_{g}$ of the monolayer graphene can be characterized by the Kubo formula [21] as a sum of two terms: $\sigma_{g}=\sigma_{\mathrm{intra}}+\sigma_{\mathrm{inter}}$. The first term corresponds to the intra-band electron-photon scattering is expressed as:

(2) $$\sigma_{\mathrm{intra}}=i\frac{e^{2}K_{B}T}{\pi^{2}\left(\omega +i\tau^{-1}\right)}\left[\frac{\mu_{c}}{K_{B}T}+2\ln(\exp(-\frac{\mu_{c}}{K_{B}T})+1)\right]$$

The second term corresponds to the inter-band transition contribution and is expressed as:

(3) $$\sigma_{\mathrm{inter}}=i\frac{e^{2}}{4\pi \hbar^{2}}\ln\left[\frac{2\left|\mu_{c}\right|-\hbar (\omega +i\tau^{-1})}{2\left|\mu_{c}\right|+\hbar (\omega +i\tau^{-1})}\right]$$

where *e* is the electron charge, $K_{B}$ is the Boltzmann’s constant, T is the temperature, $\mu_{c}=\hbar v_{f}{\left(\pi n_{s}\right)}^{1/2}$ is the chemical potential, ω is the angular frequency, ℏ is the reduced Planck’s constant, and $\tau =\mu \mu_{c}/(ev_{f}^{2})$ stands for the momentum relaxation time due to charge carrier scattering. The Fermi velocity $v_{f}$ is set at 10^6^ m/s, and the carrier mobility of graphene μ is assumed as 4 m^2^/V·s at T=300 K [22,23]. In particular, the doping level of graphene $n_{s}$ shows a linear dependence on the external gate voltage as $n_{s}=\varepsilon_{p}\varepsilon_{0}V_{b}/(eh)$ [24], where $\varepsilon_{p}$ and h are the relativity permittivity and thickness of PMMA, respectively, and $V_{b}$ is the external voltage.

**Figure 2:**
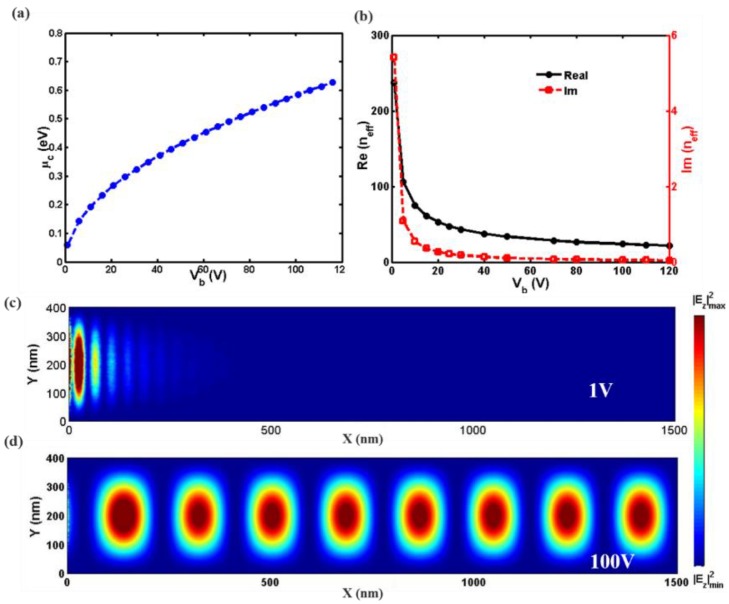
(**a**) Dependence of chemical potential $\mu_{c}$ on the bias voltage with h = 50 nm; (**b**) The real and imaginary parts of the effective index of graphene-based surface plasmons (SPs) as functions of the gate voltage $V_{b}$. Distributions of |*E_z_*|^2^ in the *x*-*y* plane of the waveguide (*h* = 50 nm, *f* = 30 THz), in the (**c**) OFF and (**d**) ON states.

The above description indicates that we can control the optical conductivity of graphene by adjusting the external applied voltage or dielectric thickness. To demonstrate, we applied a voltage bias to the graphene/PMMA/Si, as schematically shown in Figure 1f. The dependence of the chemical potential on the bias voltage is plotted Figure 2a, presenting that an increased bias voltage (from 0 V to 120 V) results in a monotone increase of chemical potential.

As is well known, graphene sheets can behave optically similar to ultrathin metallic films; therefore, our approach could realize terahertz switches by utilizing graphene-based SPs. The influence of the Si substrate on the SP dispersion can be neglected in our proposed architectures due to the thick PMMA layer [25]. Therefore, the dispersion relation of graphene-based SPs can be derived as follows [26,27,28,29]:

(4) $$\frac{\varepsilon_{c}}{k_{0}\sqrt{n_{eff}^{2}-\varepsilon_{c}}}+\frac{\varepsilon_{p}}{k_{0}\sqrt{n_{eff}^{2}-\varepsilon_{p}}}+\frac{i\sigma_{g}}{\omega \varepsilon_{0}}=0$$

where $k_{0}=2\pi /\lambda$ is the free-space wave vector of light, λ is the incident wavelength in vacuum, $n_{eff}$ is the effective refractive index of the SP mode, $\varepsilon_{c}$ denotes the relative permittivity of air, and $\varepsilon_{p}$ is the relative permittivity of PMMA. Here, we set $\varepsilon_{c}=1$ and $\varepsilon_{0}=8.854\times 10^{-12}$. According to Equation (4), the surface conductivity of graphene determines the effective refractive index $n_{eff}$ of the SP mode, which is extremely sensitive to h and $V_{b}$. Generally, the effective refractive index decreases with increasing $V_{b}$ due to the enlarged electric field. In the terahertz region, the surface conductivity of graphene can be simplified into the Drude-like form [12,19]. From the above equations, the real part of the effective refractive index can be approximated as:

(5) Re(neff)=ωπℏ(εd+1)/{η0e2vf[πεdε0Vb/(eh)]0.5}

Note that the above dispersion equation is obtained only considering the influence of external voltage $V_{b}$ [25]. Figure 2b shows that the effective refractive index of the graphene-based SPs can be electrically modified. The real part of the effective mode index decreases as the voltage bias $V_{b}$ increases, causing the reduction of SPs’ mode confinement. In addition, the imaginary part also decreases sharply as $V_{b}$ increases, which indicates that the propagation loss of SPs decreases [30,31,32,33,34]. Hence, it is expected that the longer propagation distance of the SPs occurs at a larger bias voltage. 

To demonstrate the effectiveness of our designs, numerical simulations were performed using the finite element method. Noting that during the experimental fabrication, the chemical treatment and crystal growth direction may result in imperfections in the graphene, such as the formation of polycrystalline graphene from folding defects. This would improve the light absorption of graphene [35,36]. However, we only numerically investigate the ideal single layer graphene for simplicity. A plane wave at a frequency of 30 THz is normally incident from the −*x* direction. Graphene-based surface plasmons (GSPs) will be excited so that the electromagnetic energy is strongly concentrated near graphene and propagates along the graphene layer. Perfect matched layers have been used to absorb any reflected and transmitted field. As shown in Figure 2c, when the graphene is biased to $V_{b}=1\;V$, the SPs wave propagate a very short distance and no signal reaches the output port. This is labeled as the OFF or “0” state. As shown in Figure 2d, while the bias voltage increases to $V_{b}=100$ V, the SPs have a long wave propagation distance and can reach the output port. This is labeled as the ON or “1” state. Therefore, the propagation of the SPs wave can be switched ON or OFF (“1” or “0”) by modifying the bias voltage. To optimize our proposed optical switch, we investigated the dependence of the graphene waveguide and the working bandwidth was about 0.7 µm (not shown here). Accordingly, we chose the center wavelength to be 10 um. Subsequently, we demonstrated the potential of the proposed scheme for active switching in practical applications by designing several switches.

## 3. Results and Discussion

### 3.1. Y-Branch Switch

**Figure 3:**
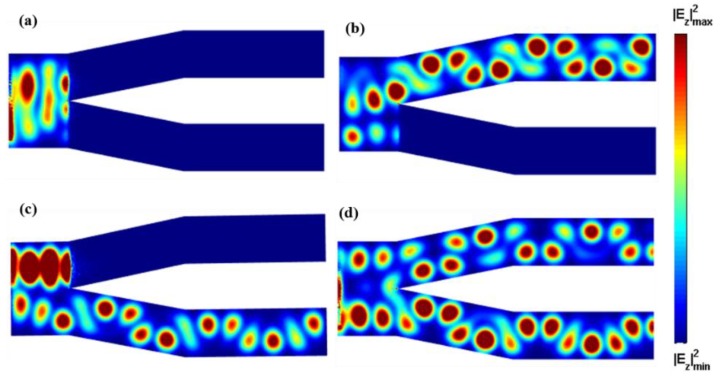
Distributions of |*E_z_*|^2^ in the *x*-*y* plane of the Y-branch switch, in the output logical state of (**a**) “00”; (**b**) “01”; (**c**) “10”; and (**d**) “11”.

Based on the structure presented above, we designed a Y-branch (1 × 2) plasmonic switch, as shown in Figure 1a. The geometry parameters of the input block are W = 800 nm and *L*_1_ = 500 nm. The output block consists of two identical output ports with *L*_1_ = 1000 nm and *L*_2_ = 1500 nm. The bias voltage of the top graphene layer of the input block is kept at $V_{b}=100$ V. Figure 3a shows the electric field distributions when both output blocks are biased to $V_{b}=1\;V$. It is easy to see that both branch arms of the Y-block are OFF, corresponding to the “00” logic state. Figure 3b,c illustrates the electric field distributions of the “01” and “10” states, respectively, when one of the output blocks is biased to $V_{b}=100$ V and the other to $V_{b}=1$ V. When both arms are biasing with $V_{b}=100$ V, the switching is in the “11” state, as depicted in Figure 3d.

In order to investigate the performance of the Y-branch (1 × 2) switch, Table 1 summarizes the transmission coefficient of each output channel at different switch states, calculated by dividing the energy of the output side by that of the input side. For the high external voltage ($V_{b}=100$ V) at the output branch, which is “1”, the transmission coefficient is about 10%. For the low external voltage ($V_{b}=1$ V) at the output branch of transmission, the corresponding transmission coefficient is zero.

### 3.2. X-Branch Switch

**Figure 4:**
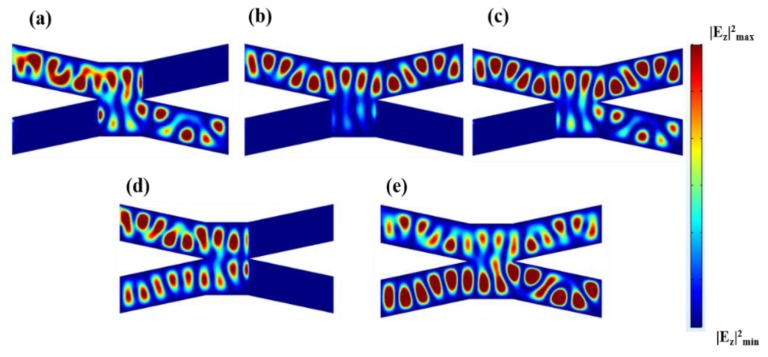
Distributions of |*E_z_*|^2^ in the *x*-*y* plane of the X-branch switch. When the input branches “I” and “II” are ON and OFF, respectively, corresponding to input state of “10”, the output branches are in the logical states of (**a**) “01”; (**b**) “10”; and (**c**) “11”. When the input branches “I” and “II” are both ON, corresponding to input state of “11”, the output branches are in the logical states of (**d**) “00” and (**e**) “11”.

Figure 1b schematically shows an X-branch (2 × 2) switch, in which the two input branches are “I” and “II”, and two output branches are “III” and “IV”. The geometry parameters were chosen as: *W* = 400 nm, *h* = 50 nm, *L*_1_ = 1000 nm, *L*_2_ = 500 nm, *d*_1_ = 200 nm. Due to the symmetry of the structure, we first took the example of branch “I” and “II” as the ON and OFF states, respectively. The states of “III” and “IV” can be well controlled due to the electrical tunability of graphene, as demonstrated in Figure 4a–c. When the external voltage of branch “III” is $V_{b}=100$ V, the input electromagnetic field from branch “I” can propagate along the output branch “III”. On the contrary, when the external voltage of branch “III” is set to $V_{b}=1$ V, it cannot propagate on this branch. In addition, we can see a similar response for branch “IV”. We also explored the condition when both branches “I” and “II” are in the ON state. Figure 4d,e further demonstrate that the output state can be switched to “00“ and “11“.

Table 2 summarizes the transmission coefficients of the output channels “III” and “IV” at different states. When a high voltage is applied at any output branch, the graphene-based SPs have a low propagation loss and high confinement. Therefore, the transmission coefficient of this output port will be at a high level, reaching the highest value of almost 40%. In contrast, when we set a low voltage at any output branch, the graphene-based SPs have an extremely high propagation loss. Thus, the transmission coefficient of the port is zero, corresponding to the OFF state, and there is no energy transmitted through this output branch.

### 3.3. Single-Input Three-Output Switch

**Figure 5:**
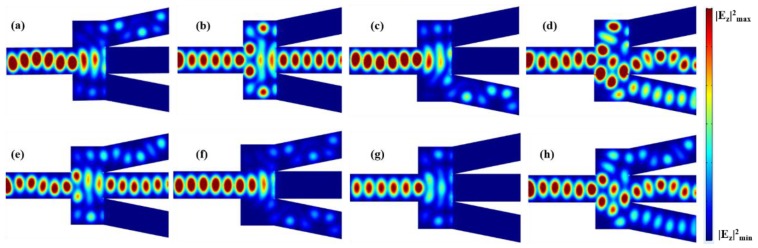
Distributions of |*E_z_*|^2^ in the *x*-*y* plane of the single-input three-output switch, in the output logical states of (**a**) “100”; (**b**) “010”; (**c**) “010”; (**d**) “011”; (**e**) “110”; (**f**) “101”; (**g**) “000” and (**f**) “111”.

Figure 1c shows the 1 × 3 switch, where the single input port is labeled as “I”, and three output branches are labeled as “II”, “III”, and “IV”. The geometry parameters were chosen as: *W* = 400 nm, *W*_1_ = 200 nm, *h* = 50 nm, *d*_1_ = 200 nm, *L*_1_ = 1000 nm, *L*_2_ = 500 nm. In Figure 5a–c, we obtained the state in which one of the output branches is ON and the other two are OFF. Specifically, we applied a high voltage of $V_{b}=100$ V at one arbitrary output branch and a low voltage of $V_{b}=1$ V at the other two output branches. In Figure 5a, the output branch “II” is ON and branches “III”, “IV” are OFF. Therefore, the input electromagnetic field can only pass through the output branch “II” and is prohibited in the other two output branches, which means that the total output state is displayed as “100”. Similarly, Figure 5b,c display the output states of “010” and “001”, respectively. In Figure 5d–f, we found that one of the output branches is OFF and the other two are ON. Specifically, we applied a low voltage of $V_{b}=1$ V at one arbitrary output branch and a high voltage of $V_{b}=100$ V at the other two output branches. For example, in Figure 5d, the branch “II” is OFF and the branches “III”, “IV” are ON. Therefore, the input electromagnetic field is prohibited in branch “II” and could pass through branches “III” and “IV”. This means that the total output state is displayed as “011”. Similarly, Figure 5e,f display the output states of “110” and “101”, respectively. Moreover, in Figure 5g,h, we showed the state in which all output branches are OFF and ON, meaning that the input electromagnetic is prohibited and can propagate through all output branches, respectively. The output states of Figure 5g,h are “000” and “111”, respectively.

Table 3 summarizes the transmission coefficients of output branches “II”, “III”, and “IV” for all output states. When we set a low voltage at the output branch, we observed that the transmission coefficient is zero, which is defined as the OFF state. When we set a high voltage at the output branch, we observed that the transmission coefficient is high, which is defined as the ON state. Note that, although there is a transmission coefficient of about 2%, it still much higher than the OFF state (zero). Thus, we can still use this structure as an optical switch.

### 3.4. Two-Input Three-Output Switch

**Figure 6:**
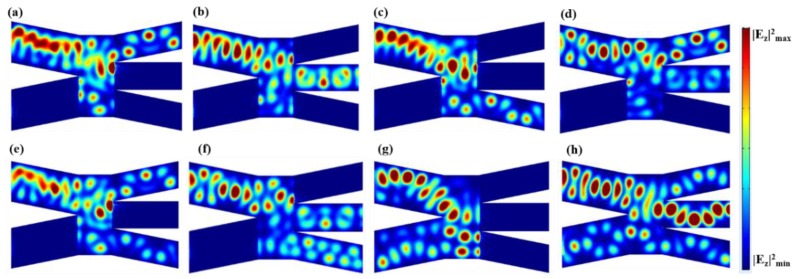
Distributions of |*E_z_*|^2^ in the *x*-*y* plane of the two-input three-output switch. When the input branches “I” and “II” are ON and OFF, respectively, corresponding to input states of “10”, the output branches are in the logical states of (**a**) “100”; (**b**) “010”; (**c**) “001”; (**d**) “110”; (**e**) “101”; and (**f**) “011”. When the input branches “I” and “II” are both ON, corresponding to the input state of “11”, the output branches are in the logical states of (**g**) “000” and (**h**) “111”.

We know that multiple-input multiple-output technology is one of the key features to extend the capacity of a communication system. Therefore, we further investigated a two-input three-output (2 × 3) switch, as schematically shown in Figure 1d. The geometry parameters were chosen as: *W*_0_ = 600 nm, *W*_1_ = 400 nm, *W*_2_ = 200 nm, *h* = 50 nm, *d*_1_ = 200 nm, *L*_1_ = 1000 nm, *L*_2_ = 500 nm. The stimulated SP wave can be coupled into the output branch. The biasing conditions determine whether the output branches are ON or OFF. Because of the structural symmetry, we took the example of input branches “I” and “II” as ON and OFF, respectively, corresponding to the input state of “10”. Figure 6a–f show the electric field distributions when we control the external voltage to determine whether the electromagnetic field can pass through the output branches “III”, “IV”, and “V”, obtaining corresponding output states. In Figure 6a–c, the output branches are in the logical states of “100”, “010”, and “001”, respectively. We further obtained the output states “110”, “101”, and “011” in Figure 6d–f, respectively. In Figure 6g,h, we demonstrate that the three output branches can be OFF and ON at the same time, corresponding to the output states of “000” and “111”, respectively.

Table 4 summarizes the transmission coefficients of the output channels “III”, “IV”, and “V” at different logical states. When we set a low voltage ($V_{b}=1$ V) at each output branch, corresponding to the OFF state, the transmission coefficient is zero. When we set a high voltage ($V_{b}=100$ V) at each output branch, corresponding to the ON state, the transmission coefficient can be much higher. Although there is a lowest transmission coefficient of about 2%, it still relatively higher than that of the OFF state (zero). Therefore, we can still consider this simple transmission line as a 2 × 3 optical switch.

### 3.5. Two-Input Four–Output Switch

**Figure 7:**
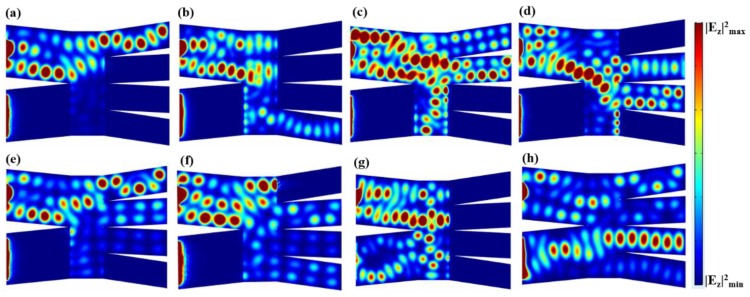
Distributions of |*E_z_*|^2^ in *x*-*y* plane of the two-input four-output switch. When the input branches “I” and “II” are ON and OFF, respectively, corresponding to the input state of “10”, the output branches are in the logical states of (**a**) “1000”; (**b**) “0001”; (**c**) “1100”; (**d**) “0110”; (**e**) “0111” and (**f**) “0111”. When the input branches “I” and “II” are both ON, corresponding to input state of “11”, the output branches are in the logical states of (**g**) “0000” and (**h**) “1111”.

We applied the proposed structure to a two-input four-output (2 × 4) switch, as shown in Figure 1e. The geometry parameters were chosen as: *W*_0_ = 800 nm, *W*_1_ = 200n m, *W*_2_ = 400 nm, *W*_3_ = 100 nm, *h* = 50 nm, *d*_1_ = 200 nm, *L*_1_ = 1000 nm, *L*_2_ = 500 nm. Figure 7 shows the electric field distribution of the switch at different input and output states. Firstly, we considered the case in which the input branches “I” and “II” are ON and OFF, respectively. Figure 7a–f show the electric field distributions when we control the external voltage to determine whether the electromagnetic field can pass through the output branches “III”, “IV”, “V”, and “VI”. For the case in which only one output branch is ON, we took the example of an electric field that is controlled to propagate in either branch “III” or “VI”. Therefore, the corresponding output states of “1000” and “0001” are achieved, as shown in Figure 7a,b, respectively. For the case in which two output branches are ON, we took the example of an electric field that is controlled to propagate in either branches “III”, “IV” or “IV”, “V”. This corresponds to the output states of “1100” and “0110”, as shown in Figure 7c,d, respectively. We further investigated the case in which three output branches are ON. As examples, we showed that the electromagnetic field can pass through either branches “III”, “IV”, “V” or “IV”, “V”, “VI”, corresponding to the output states of “1110” or “0111”, as shown in Figure 7e,f, respectively. In Figure 7g,h, we further demonstrated that the four output branches can be OFF and ON at the same time, corresponding to output states of “0000” and “1111”, respectively.

Table 5 summarizes the transmission coefficients of output channels “III”–“VI”. When an output branch is OFF, no input energy can propagate in it and the transmission coefficient is zero. When an output branch is ON, the input energy can pass through it due to the low loss of graphene SPs. Even though the transmission coefficient is only about 2%, we can still consider the structure to be an optical switch. In future study, we will continue to explore the transmittance efficiency issue to further optimize the present work. 

## 4. Conclusions

In summary, we have theoretically and numerically investigated multiple-input multiple-output switches. The applied gate voltage can be employed to tune the conductivity of graphene to control whether it can be propagated at the graphene surface. Moreover, each branch can realize an ON/OFF state by adjusting the external voltage. Thus, we designed a Y-branch plasmonic waveguide to realize a single-input two-output switch by biasing an appropriate voltage at the graphene layer and Si substrate. We also designed X-branch (2 × 2), single-input three-output (1 × 3), two-input three-output (2 × 3), and two-input four-output (2 × 4) switches to realize multiple-input multiple-output transmission. All of the proposed structures together demonstrate good switching performances with high controllability and subwavelength confinement of electromagnetic waves. These structures can be widely used in the manipulation of information propagation and optical switches, and have far-reaching significance.

## Figures and Tables

**Table 1:** Transmission coefficients of the Y-branch switch at different logical states.

Output Signal		Transmission Coefficient (%)	
Output II	Output III	Output II	Output III
0	0	0	0
1	0	14.418	0
0	1	0	13.872
1	1	9.589	13.042

**Table 2:** Transmission coefficients of the X-branch switch at different logical states.

Input Signal		Output Signal		Transmission Coefficient (%)	
Input I	Input II	Output III	Output IV	Output III	Output IV
1	0	0	1	0	9.775
1	0	1	0	27.221	0
1	0	1	1	36.785	13.004
1	1	0	0	0	0
1	1	1	1	27.016	49.695

**Table 3:** Transmission coefficients of the single-input three-output switch at different logical states.

Input Signal	Output Signal			Transmission Coefficient (%)		
Input I	Output II	Output III	Output IV	Output II	Output III	Output IV
1	1	0	0	5.557	0	0
1	0	1	0	0	58.527	0
1	0	0	1	0	0	3.848
1	0	1	1	0	70.106	2.011
1	1	1	0	2.215	63.966	0
1	1	0	1	4.011	0	3.145
1	0	0	0	0	0	0
1	1	1	1	1.743	54.373	3.835

**Table 4:** Transmission coefficients of the two-input three-output switch at different logical states.

Input Signal		Output Signal			Transmission Coefficient (%)		
Input I	Input II	Output III	Output IV	Output V	Output III	Output IV	Output V
1	0	1	0	0	6.913	0	0
1	0	0	1	0	0	20.86	0
1	0	0	0	1	0	0	1.183
1	0	1	1	0	1.391	13.566	0
1	0	1	0	1	1.29	0	4.502
1	0	0	1	1	0	19.722	9.884
1	1	0	0	0	0	0	0
1	1	1	1	1	6.281	30.536	7.833

**Table 5:** Transmission coefficients of the two-input four-output switch at different logical states.

Control Signal		Output Signal				Transmission Coefficient (%)			
Input I	Input II	Output III	Output IV	Output V	Output VI	Output III	Output IV	Output V	Output VI
1	0	1	0	0	0	0.157	0	0	0
1	0	0	0	0	1	0	0	0	0.172
1	0	0	1	1	0	0	0.105	1.394	0
1	0	1	1	0	0	1.688	0.161	0	0
1	0	1	1	1	0	0.114	0.279	0.01	0
1	0	0	1	1	1	0	0.317	0.012	0.026
1	1	0	0	0	0	0	0	0	0
1	1	1	1	1	1	0.0805	0.376	0.486	0.0886

## References

[1] Zhou J., Wong W., Pun E., Shen Y. (2006). Fabrication of low loss optical waveguides with a novel thermo-optical polymer material. Opt. Appl..

[2] Vicente C., Lima P., Bermudez V., Carlos L., André P., Ferreira R. (2014). Fabrication of low-cost thermo-optic variable wave plate based on waveguides patterned on di-ureasil hybrids. Opt. Express.

[3] Stolte R., Ulrich R. (1995). Electro-optic and thermo-optic measurements of birefringence of LiNbO_3_ waveguides. Opt. Lett..

[4] Guo Z., Xu H., Guo K., Shen F., Zhou H., Zhou Q., Gao J., Yin Z. (2018). High-efficiency visible transmitting polarizations devices based on the GaN metasurface. Nanomaterials.

[5] Yin Z., Chen F., Zhu L., Guo K., Shen F., Zhou Q., Guo Z. (2018). High-efficiency dielectric metasurfaces for simultaneously engineering polarization and wavefront. J. Mater. Chem. C.

[6] Ozbay E. (2006). Plasmonics: Merging photonics and electronics at nanoscale dimensions. Science.

[7] Ge C., Guo Z., Sun Y., Shen F., Tao Y., Zhang J., Li R., Luo L. (2016). Spatial and spectral selective characteristics of the plasmonic sensing using metallic nanoslit arrays. Opt. Commun..

[8] Zheng Y.B., Yang Y.W., Jensen L., Fang L., Juluri B.K., Flood A.H., Weiss P.S., Stoddart J.F., Huang T.J. (2009). Active molecular plasmonics: Controlling plasmon resonances with molecular switches. Nano Lett..

[9] Koppens F.H., Chang D.E., Garcia de Abajo F.J. (2011). Graphene plasmonics: A platform for strong light–matter interactions. Nano Lett..

[10] Gusynin V.P., Sharapov S.G., Carbotte J.P. (2006). Magneto-optical conductivity in graphene. J. Phys. Condens. Matter.

[11] Chen J., Jang C., Xiao S., Ishigami M., Fuhrer M.S. (2008). Intrinsic and extrinsic performance limits of graphene devices on SiO_2_. Nat. Nanotechnol..

[12] Chen P., Alu A. (2011). Atomically thin surface cloak using graphene monolayers. ACS Nano.

[13] Ju H., Bing W., Jiang Y., Gao Z. (2012). Beam-scanning planar lens based on graphene. Appl. Phys. Lett..

[14] Yarahmadi M., Moravvej-Farshi M., Yousefi L. Compact low power graphene-based Y-branch THz switch. Proceedings of the Third Conference on IEEE Millimeter-Wave and Terahertz Technologies.

[15] Yarahmadi M., Moravvej-Farshi M.K., Yousefi L. (2015). Subwavelength graphene-based plasmonic THz switches and logic gates. IEEE Trans. Terahertz Sci. Technol..

[16] Wu X., Tian J., Yang R. (2017). A type of all-optical logic gate based on graphene surface plasmon polaritons. Opt. Commun..

[17] Peng C., Li J., Liao H., Li Z., Sun C., Chen J., Gong Q. (2018). Universal linear-optical logic gate with maximal intensity contrast ratios. ACS Photonics.

[18] Shi B., Cai W., Zhang X., Xiang Y., Zhan Y., Geng J., Ren M., Xu J. (2016). Tunable band-stop filters for graphene plasmons based on periodically modulated graphene. Sci. Rep..

[19] Hao R., Peng X., Li E., Xu Y., Jin J., Zhang X., Chen H. (2015). Improved slow light capacity in graphene-based waveguide. Sci. Rep..

[20] Zhang F.M., He Y., Chen X. (2009). Guided modes in graphene waveguides. Appl. Phys. Lett..

[21] Christensen J., Manjavacas A., Thongrattanasiri S., Koppens F.H., García de Abajo F.J. (2011). Graphene plasmon waveguiding and hybridization in individual and paired nanoribbons. ACS Nano.

[22] Bolotin K., Sikes K., Jiang Z., Klima M., Fudenberg G., Hone J., Kim P., Stormer H. (2008). Ultrahigh electron mobility in suspended graphene. Solid State Commun..

[23] Qiu W., Liu X., Zhao J., Huang Y., Chen H., Li B., Wang J., Pan J.Q. (2015). Ultrabroad band rainbow capture and releasing in graded chemical potential distributed graphene monolayer. Plasmonics.

[24] Novoselov K.S., Geim A.K., Morozov S.V., Jiang D.A., Zhang Y., Dubonos S.V., Grigorieva I.V., Firsov A.A. (2004). Electric field effect in atomically thin carbon films. Science.

[25] Lu H., Zeng C., Zhang Q., Liu X., Hossain M.M., Reineck P., Gu M. (2015). Graphene-based active slow surface plasmon polaritons. Sci. Rep..

[26] Jablan M., Buljan H., Soljačić M. (2009). Plasmonics in graphene at infrared frequencies. Phys. Rev. B.

[27] De Oliveira R.E., De Matos C.J. (2015). Graphene based waveguide polarizers: In-depth physical analysis and relevant parameters. Sci. Rep..

[28] Luo L., Wang K., Guo K., Shen F., Zhang X., Yin Z., Guo Z. (2017). Tunable manipulation of terahertz wavefront based on graphene metasurfaces. J. Opt..

[29] Wang J., Song C., Hang J., Hu Z.D., Zhang F. (2017). Tunable Fano resonance based on grating-coupled and graphene-based Otto configuration. Opt. Express.

[30] Wang W., Meng Z., Liang R., Chen S., Ding L., Wang F., Liu H., Wei Z. (2018). A dynamically tunable plasmonic multi-functional device based on graphene nano-sheet pair arrays. Opt. Commun..

[31] Luo L., Wang K., Ge C., Guo K., Shen F., Yin Z., Guo Z. (2017). Actively controllable terahertz switches with graphene-based nongroove gratings. Photonics Res..

[32] Wang J., Wang X., Hu Z.D., Zheng G., Zhang F. (2017). Peak modulation in multi-cavity-coupled graphene-based waveguide system. Nanoscale Res. Lett..

[33] Gómez-Díaz J.S., Perruisseau-Carrier J. (2013). Graphene-based plasmonic switches at near infrared frequencies. Opt. Express.

[34] Yang J., Xin H., Han Y., Chen D., Zhang J., Huang J., Zhang Z. (2017). Ultra-compact beam splitter and filter based on a graphene plasmon waveguide. Appl. Opt..

[35] Lim G., Kihm K.D., Kim H.G., Lee W., Lee W., Pyun K.R., Cheon S., Lee P., Min J.Y., Ko S.H. (2018). Enhanced thermoelectric conversion efficiency of CVD graphene with reduced grain sizes. Nanomaterials.

[36] Kim K., Lee H., Johnson R.W., Tanskanen J.T., Liu N., Kim M.G., Pang C., Ahn C., Bent S.F., Bao Z. (2014). Selective metal deposition at graphene line defects by atomic layer deposition. Nat. Commun..

